# On the Melksham Chalybeate and Saline Aperient Spa

**Published:** 1813-10

**Authors:** G. S. Gibbes

**Affiliations:** Laura-Place, Bath


*For the Medical and Physical Journal.*

### *On the* MELKSHAM CHALYBEATE *and* SALINE APERIENT SPA,

*Situated near the Great London Road, about half a mile from the Town of Melksham, and twelve miles from Bath.*

AS the following short analysis of this water, when joined to its known medicinal qualities, certainly ranks it with the saline purgative waters of Cheltenham and Leamington, it cannot be deemed improper to apprize the public of the existence of so valuable a spring in this neighbourhood. The medicinal effects of this water, clearly point out that it is a saline purgative; but its taste, although strongly saline, and greatly resembling the sea and other waters of the same description, is more unexceptionable; and, from being composed of many substances, which are combined in the great laboratory of Nature, whereby the exact balance is probably preserved under a triple, or even a quadruple alliance between the several salts contained in it, this water, like others of a similar kind, produces an aperient effect, with the smallest possible expenditure of purgative ingredients, and without that intenseness of saline impression, and that repulsion of the taste, which take place in the artificial solutions of the neutral salts.

The spring rises in a field near Melksham, from nearly the top of a mound of earth, which was formed about fifty years ago of the materials which had been dug out in sinking a shaft for the purpose of seeking for coal. After penetrating to a great depth, the miners came to a very hard rock, on piercing through which, this water rushed in upon them, and was so abundant that the scheme for finding coal was entirely abandoned. The shaft was filled up with timber and earth, and the spring has ever since continued to flow above the original level of the field. At this height it produces a pint of water in three quarters of a minute, from which circumstance it is presumed that at some depth an almost indefinite quantity of it might be obtained.

Many people in the neighbourhood have for some time past experienced salutary effects from this water as a medicine, their notice having been attracted to its peculiar qualities by the consequences to the cattle that drank it, and by the frequent visits of the wood pigeons to the spring.

It was owing to the observations made by Mr. Flower of its saline quality, and to the advantage which Mr. Phillips, a gentleman of Melksham, derived from using this instead of Cheltenham water, that it has this summer been brought into notice; and in compliance with a request made by them, Mr. J. Long and Mr. Bruges, I have thus brought my observations and experiments before the public.

I accurately weighed four ounces of this water in an evaporating earthen vessel, which I had previously put in equipoise, with correspondent weights in the other scale; when, gradually evaporating the water, I found, after placing a four ounce weight in the scale with the vessel, that it took twenty-one grains in the other scale to restore the exact equilibrium. The balance is a most excellent one, and nothing occurred that could detract from the accuracy of the experiment. Twenty-one grains in four ounces, allowing twelve ounces by weight to the pint, make sixty-three grains in a pint, and five hundred and four in a gallon, or five hundred and fifty-two grains in a gallon or more if brought to sixteen ounces avoirdupoise in a pint. As I brought the evaporation quite to dryness before a large fire, and as many saline substances found in mineral waters contain, when crystallized, from 30 to 50 per cent. of water, this quantity far exceeds the estimate given by Dr. Fothergill of the contents of a gallon of Cheltenham water, namely, 555 grains of crystallized salts.

I am also authorized by Dr. Sims to state, that he procured from a beer gallon of this water 955 grains of crystallized salts, equal to 782 grains in the wine gallon, which is at least 227 grains more than Dr. Fothergill found in the same quantity of Cheltenham water, and allowing about 40 per cent. for the water of crystallization, is in exact coincidence with my experiment.

The tincture of litmus was not reddened by it; but litmus which had been reddened by a slight acid, had its color restored on the addition of the water.

Salts, both earthy and saline, with sulphuric acid, are contained in this water; for a precipitation ensues on the addition of muriate of barytes and the oxalate of ammonia. A prodigious precipitation takes place on the addition of the nitrates of mercury, and of silver; therefore the water contains a large proportion of muriatic salts, the principal of which which is the muriate of soda, or common salt. The crystallization of an immense number of cubes, when examined in the microscope, shows this fact, and points out a resemblance between this water and that of Leamington, in Warwickshire.

Although there appears to be a ferruginous precipitation after the water has stood some time, and some appearance of the kind around the source of it, yet I could not observe that either tincture of galls, or the prussiate of potash had any sensible effect upon the water when it had been removed some hours from the spring; but, at the source, although there was no indication of iron by the prussiate of potash, yet the fresh watery infusion of galls struck a purple in it, and when the calcareous earth had been precipitated by oxalate of ammonia, there was an evident purple color induced by the tincture of galls.

The slightest if any change takes place on the addition of muriate of lime; there is therefore no alkaline carbonate, which I had suspected, from the decided effect produced on the reddened litmus, though that may arise from the carbonate of lime contained in the water.

The nitrates of silver and mercury produce perfectly white precipitations, therefore the water contains no sulphur; even at the source, although there is a slight smell and taste of sulphurated hydrogene, yet the nitrate of mercury produces a perfectly white precipitation.

After the calcareous earth had been separated by the oxalates of potash and ammonia, and the clear liquor filtered, pure ammonia produced a precipitation. After carbonate of ammonia had been added to the clear filtered liquor from which all calcareous earth had been precipitated by means of the oxalate of potash, a solution of phosphate of soda produced a precipitation confirming the first experiments as indicating the presence of magnesia. It is owing generally to the muriate of magnesia, that the purgative effect of these natural saline waters is increased; indeed, the combinations of the salts contained in mineral waters, which are effected by nature, far exceed in power any artificial arrangement, either as to the quantity they contain, or as to the quality of those usually found in them.

Although this water has been so lately made the subject of inquiry, many well-attested cases occur of its efficacy in both bilious and scorbutic habits. In doses, similar to the Cheltenham and Leamington water, it acts on the bowels gently, safely, but decidedly; and I find that it neither produces heaviness on the stomach, nor in any way disagrees with the constitution. I can discover no substance from which noxious qualities can be supposed to arise; and I see no reason why it should not be resorted to, whenever it may be necessary to avoid either the trouble or expense of a journey to Cheltenham or Leamington.

As many chemical as well as medical friends have kindly communicated the result of their inquiries in respect to the qualities and medicinal properties of this water, and as their opinions have been uniformly favourable to its character, and in unison with my own, I can have no hesitation in speaking with increased confidence of the advantages which must result from its use.

The Melksham Spa water contains several substances, that are very active, and which determine the medical properties of many distinguished mineral waters. Its character is saline, and the quantity of saline ingredients is equal to that of the most celebrated springs. The salts contained in it are in their nature purgative, and therefore a constant effect on the bowels, is the action this medicinal water produces whenever it is taken in suitable doses. A countervailing property in this water arises from the presence of some iron, thereby precluding that debility which so often follows the use of the stronger purgatives.

A moderate dose, for instance, half-a-pint, will often act strongly; two half-pints seldom fail to produce a copious effect, and a pint and a half in the course of the day has proved generally a powerful dose of physic.

In doses too small to produce any action on the bowels, it passes off readily by urine, and thus it appears to combine a variety of salutary operations.

The course of the water may be persevered in without interruption for a considerable length of time, even in states of apparently great debility, without producing any inconvenience to the system. The appetite is much improved by its use; it promises the happiest effects in most of the disorders of the digestive organs; and as far as its powers have been observed, it wonderfully improves all those habits of body which are the regular attendants of indigestion. It seems calculated in a superior degree to relieve and remove the bad consequences resulting from bilious obstruction, and to restore to the function of the liver in secreting bile, a due and healthy regularity.

There is also a strong chalybeate spring in the same field.

The eligibility of the situation is unquestionable, it being close to the neat and respectable town of Melksham, and only twelve miles from the city of Bath; and from the above experiments and observations, we may presume that an excellent preparative or auxiliary to a course of the Bath waters, may be found in the Melksham Saline Aperient.

*Laura-Place, Bath,* G. S. GIBBES, M.D.

*August* 20, 1813.

